# Nitrogen fixation may not alleviate stoichiometric imbalances that limit primary production in eutrophic lake ecosystems

**DOI:** 10.1002/ecy.4516

**Published:** 2025-01-24

**Authors:** Isabelle M. Andersen, Jason M. Taylor, Patrick T. Kelly, Alexa K. Hoke, Caleb J. Robbins, J. Thad Scott

**Affiliations:** ^1^ Department of Biology Baylor University Waco Texas USA; ^2^ US Department of Agriculture, Agricultural Research Service, National Sedimentation Laboratory Oxford Mississippi USA; ^3^ Department of Biology Rhodes College Memphis Tennessee USA; ^4^ Institute of Arctic Biology, University of Alaska Fairbanks Fairbanks Alaska USA; ^5^ Present address: Office of Great Waters, Wisconsin Department of Natural Resources La Crosse Wisconsin USA

**Keywords:** ecosystem stoichiometry, mesocosm, nitrogen, nitrogen fixation, nutrient limitation, phosphorus

## Abstract

Ecosystem‐scale primary production may be proximately limited by nitrogen (N) but ultimately limited by phosphorus (P) because N_2_ fixation contributes new N that accumulates relative to P at ecosystem scales. However, the duration needed to transition between proximate N limitation and ultimate P limitation remains unknown for most ecosystems, including lakes. Here we present the results of a fully replicated, multi‐annual lake mesocosm experiment that permitted full air‐water‐sediment interactions that mimicked lake ecosystem ecology. We manipulated N supply relative to P to achieve a gradient of N:P stoichiometry. Despite N_2_ fixation contributing as much as 80% of reactive N in the low N treatments, phytoplankton biomass in these treatments was not different from the unfertilized controls. This suggests that primary production remained N limited in the lowest N treatments, even when N_2_ fixation was substantial. Although fixed N inputs reduced the N imbalance relative to P in the low N treatments seasonally, fixed N did not accumulate over multiple years. Additionally, reactive N did not readily accumulate in the high N treatments. Instead, water column stoichiometry was proportional to the experimental N and P additions, suggesting a strong influence from external nutrient loading. Thus, we found no evidence that N accumulation from N_2_ fixation was sufficient to trigger a transition to ultimate P limitation seasonally or across our 3‐year experiment. Rather, our results indicate that proximate N limitation perpetuates in eutrophic lakes, likely due to N export being proportional to its inputs. These findings offer new insight regarding the biogeochemical controls on ecosystem stoichiometry and their influence on the timeframe for proximate N limitation and ultimate P limitation in freshwater, marine, and terrestrial ecosystems.

## INTRODUCTION

The mechanisms by which nitrogen (N) or phosphorus (P) limit ecosystem‐scale primary production are controlled by biogeochemical processes over variable timescales (Vitousek & Howarth, 1991). Tyrrell (1999) hypothesized that N supply *proximately* controls ocean primary production over observable timescales from hours to years, but P supply *ultimately* constrains primary production by controlling biological N_2_ fixation, the transformation of atmospheric N_2_ into biologically reactive N (Marcarelli et al., 2022). Presumably, N_2_ fixation can slowly increase the ecosystem N pool and fuel increased primary productivity over centuries to millennia. Thus, P can be considered the ultimate limiting nutrient and N the proximate limiting nutrient for the world's oceans. Vitousek et al. (2010) applied the ultimate and proximate limiting nutrient concept to terrestrial ecosystems but emphasized that changes to environmental and ecosystem functions in response to nutrient imbalance can provide direct evidence for the ultimate limiting nutrient over relatively short experimental periods. For example, fire tends to increase P availability and decrease N availability in terrestrial ecosystems, which can increase N_2_ fixation rates in free‐living soil bacteria (Yelenik et al., 2013) or increase the abundance of tree species capable of supporting symbiotic N_2_‐fixers (Wong et al., 2020). Thus, terrestrial ecosystems respond to disturbances in N and P supply stoichiometry through changes to ecosystem structure or function that result in long‐term changes in N availability relative to P (Vitousek, 2004).

Both Tyrrell's and Vitousek's ultimate and proximate limiting nutrient concepts relied on earlier evidence from the Canadian Experimental Lakes Area (ELA), where within just a few years of whole‐lake ecosystem fertilization experiments, a low N:P supply shifted phytoplankton community dominance to cyanobacterial N_2_‐fixers (Schindler, 1977). Long‐term continuation of this experiment showed that N concentrations increased over the first two decades as fertilizer N and fixed N accumulated and recycled between the sediments and water column (Schindler et al., 2008). After two decades of N and P fertilization, N fertilization was halted and P fertilization continued for another two decades. Despite increased N_2_ fixation during this P‐only fertilization period, lake N concentrations slowly declined (Scott & McCarthy, 2010), but the lake remained eutrophic (Schindler, 2012). Interpreting these complex results and their implication for freshwater eutrophication management has been controversial (Paerl et al., 2016; Schindler et al., 2016). Although N_2_ fixation provides a clear mechanism for an ecosystem to gain reactive N, ecological constraints on N_2_ fixers (Grover et al., 2019, 2022) can create a ceiling on these gains, and the competing process of denitrification provides an ecosystem N loss mechanism that may offset gains (Scott et al., 2019; Taylor et al., 2023). Thus, the eventual accumulation of fixed N relative to P may not actually occur over many years in response low N:P supply to lakes (Schindler, 1977, 2012), and instead, N_2_ fixation may only potentially meet N demand on an annual basis (Higgins et al., 2018). Collectively these results demonstrate a continued knowledge gap about how rapidly lakes respond to stoichiometric imbalances in their N and P supplies.

Nutrient supply to lakes is determined by the magnitude of nutrient load, which is the product of inflow volume and nutrient concentrations that derive from natural and anthropogenic sources. When normalized by lake surface area, P loading can range from 0.01 to 10 g P m^−2^ year^−1^ (see examples in Appendix S1). Anthropogenic sources not only increase the magnitude of nutrient loading to lakes but also alter the N:P ratio of nutrient loading (Downing & McCauley, 1992). For example, lakes with watersheds having a high proportion of row crop agriculture had higher N:P ratios in their nutrient loads than lakes in forested watersheds (Vanni et al., 2011). Conversely, lakes with watersheds containing high‐density animal agriculture had lower N:P ratios in their nutrient loads (Grantz et al., 2014). Thus, lakes show tremendous variability in their water column N:P stoichiometry (Graeber et al., 2024) and although large‐scale observational studies suggest this affects N and P limitation across thousands of lakes at continental scales (Rock & Collins, 2024), there remains a clear lack of experimental evidence. To our knowledge, no multi‐annual ecosystem‐scale experiments have rigorously tested the effect of variable N:P supply stoichiometry on the potential transition from proximate N limitation to ultimate P limitation in lakes.

Here we report the results of a 3‐year replicated experiment in which we manipulated the N and P supply in shallow lake mesocosms that permitted air‐water and sediment‐water interactions. We fertilized all mesocosms with P at 1.3 g P m^−2^ year^−1^ and varied the N fertilizer between 1.6 and 68 g N m^−2^ year^−1^, which corresponded to four different N:P ratio treatments (molar): 2.2, 16, 55, 110 (low N, medium‐low N, medium‐high N, and high N). We were interested in exploring the effects of time and N availability relative to P on the capacity of eutrophic lakes to overcome N imbalances imposed by P enrichment. We did this by observing phytoplankton biomass (as chlorophyll *a*), N_2_ fixation, and N imbalance relative to P in the water column through time and between treatments. We hypothesized three alternative outcomes, each with respective predictions that would support each hypothesis:

The shift from N limitation to P limitation occurs quickly (weeks to months) due to seasonal accumulation of N relative to P.

Phytoplankton biomass will not differ among treatments, but all treatments will have more phytoplankton biomass than the controls.

N imbalance relative to P will not differ among low N treatments and controls due to rapid balancing by N fixation.

The shift from N limitation to P limitation occurs at a moderate pace (in year[s]) due to interannual accumulation of N relative to P.

Phytoplankton biomass and N imbalance relative to P in the low N treatments would increase annually.

N_2_ fixation in the low N treatments would decrease annually.

The shift from N limitation to P limitation occurs slowly (decades) or not at all due to no interannual accumulation of N relative to P.

Phytoplankton biomass and N imbalance relative to P will be proportional to N treatment across years.

N_2_ fixation will be high and invariant in the low N treatments across years.

## METHODS

### Study site

**FIGURE 1:**
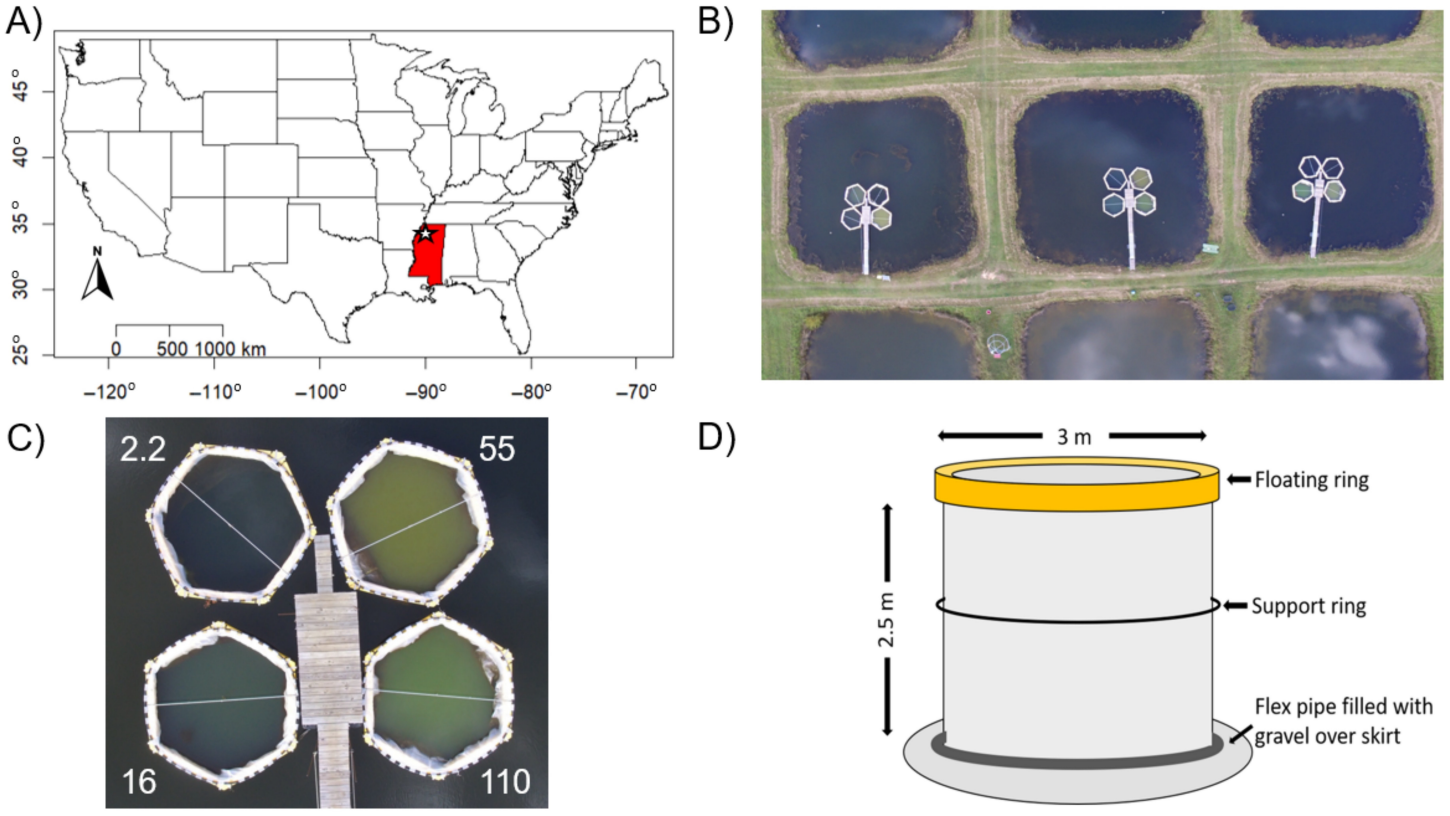
Mesocosm experiment took place in Abbeville, MS (A). Mesocosms were installed in experimental ponds (B). Four target N:P treatments were assigned to the mesocosms (C). Mesocosms separated experimental water from control pond water while still allowing for air‐water‐sediment fluxes (D). Photographs (B, C) by Mark Griffith. Diagram (D) by Jason Taylor.

This experiment was conducted as part of a long‐term aquatic mesocosm experiment at the University of Mississippi Field Station (UMFS) in Abbeville, MS, USA (34.25412 N, −89.23296 W) (Figure 1). The UMFS has a series of 48 similarly sized experimental ponds (Appendix S1). The three ponds used for this experiment all have a surface area of approximately 0.07 ha and a depth of 1.5–2 m. Twelve limnocorral mesocosms (Curry Industries Ltd., Winnipeg, Manitoba, Canada) were installed in the approximate center of three identical ponds (four per pond) in spring 2019 (Figure 1). Mesocosms were constructed as 3‐m‐diameter tubes composed of plastic sheeting that extends from a circular floating frame at the water surface to a weighted ring that anchored the mesocosm walls into the sediment (Figure 1). Each mesocosm isolated approximately 17 m^3^ of water from the surrounding pond water. The open top and bottom design created a complete air‐water‐sediment connection to best represent a lake ecosystem with an isolated water column that could be chemically independent from surrounding waters.

### Experimental design and sampling

All 12 mesocosms were fertilized with P (as NaH_2_PO_4_) at an average annual loading rate of 1.3 g m^−2^ year^−1^, and four different average annual loading rates of N (as NaNO_3_): 1.3, 9.8, 34.1, and 68.2 g m^−2^ year^−1^ (Appendix S1). The N treatments corresponded with our target N:P (molar) treatments of 2.2, 16, 55, and 110 and were selected from the range of data reported for hypereutrophic conditions in the USEPA National Lakes Assessment (Pollard et al., 2018). Each year, fertilization began in late April or early May and fertilizer was added weekly over the course of 2 months, with dosing rates being greatest in the first week and decreasing over the summer (Appendix S1). This fertilization pattern was designed to simulate natural nutrient loading due to spring precipitation patterns (Royer et al., 2006). Each mesocosm was isotopically labeled with ^15^N (as Na^15^NO_3_) to estimate N_2_ fixation by later measuring the relative inputs of fertilizer and atmospheric N in seston (Appendix S2). The three host ponds were not fertilized and served as the experimental controls.

Each mesocosm and the control ponds were sampled twice per week from May through October of 2019, once per week from June through October of 2020, and once per week from May through August of 2021. Water samples were collected at ~0.5 m depth in opaque 2 L bottles and returned to the laboratory for processing. Unfiltered water samples for total nitrogen (TN) and total phosphorus (TP) and filtered (0.45 μm) water samples for dissolved inorganic N (DIN) and dissolved inorganic P (DIP) were stored frozen at −20°C until the time of analysis. Seston samples for chl *a* analysis were captured onto a 0.7‐μm glass fiber filter (Whatman GF/F) and then were frozen at −20°C until analyzed. Seston samples for ^15^N isotopic analyses were captured onto a 0.7‐μm Qm‐A quartz fiber filter (Whatman) and frozen at −20°C until analyzed. During 2019, seston ^15^N samples were only sampled from June through July. Seston includes all matter captured on the filters but is primarily composed of phytoplankton and is often used as a proxy for phytoplankton biomass.

### Laboratory analyses

TN and TP samples were analyzed using the cadmium reduction and molybdate methods, respectively (APHA, 2005), following acid persulfate digestion. DIN and DIP samples were analyzed separately for nitrite plus nitrate N (NO_2_‐N + NO_3_‐N), ammonium (NH_4_‐N), and soluble reactive phosphate (PO_4_‐P) using the cadmium reduction, phenate, and molybdate methods, respectively (APHA, 2005). All nutrient analyses were conducted with a Lachat QuickChem 8500 Series II auto analyzer (Lachat Instruments, Hach Company, Loveland, CO, USA). Chl *a* analysis was conducted using the pheophytin‐corrected spectrophotometric method (APHA, 2005) following 90% acetone extraction (Trilogy Fluorometer, Turner Designs, San Jose, CA, USA). Sample filters for seston ^15^N were dried overnight at 60°C and wrapped in tin foil cups (Leco, 502‐186‐200) before analyses. Seston ^15^N isotopic analyses were conducted in the Baylor University Stable Isotope Laboratory using a Thermo‐Electron Delta V Advantage Isotope Ratio Mass Spectrometer (Thermo Electron, Bremen, Germany). Isotope ratios for samples were calculated relative to the atmospheric ^15^N standard and expressed in delta notation (δ^15^N‰). The fractional contribution of fixed N_2_ in seston (*F*_fix_) was estimated using a two‐source mixing model (Appendix S2).

### Calculations and statistical analyses

The N imbalance of N relative to P (N_I_, Scott et al., 2019) for all sampling events was calculated as

(1) $$N_{I}=\mathrm{TN}-\left[\mathrm{TP}\times N:P_{\mathrm{opt}}\right],$$

where TN and TP were the measured TN and TP concentrations, and N:P_opt_ was the estimated threshold for strict P limitation in seston, which we assumed to be 50 by moles (Graeber et al., 2024; Guildford & Hecky, 2000; Scott et al., 2019). Guildford and Hecky (2000) suggested that primary production would be co‐limited by N and P in lakes with a TN:TP between 20 and 50 (molar), meaning that there would still be potential for N_2_ fixation (i.e., N deficit) within that TN:TP range (Osburn et al., 2021; Wagner et al., 2023). N_I_ is an estimate of the amount of N surplus or deficit relative to P availability in any sample. A value of zero represents a sample in which TN and TP occur in identical proportions to the minimum threshold for strict P limitation (TN:TP = 50). A positive N_I_ represents an N surplus relative to P and a negative N_I_ represents an N deficit relative to P. Scott et al. (2019) showed that the magnitude of negative N_I_ (i.e., N deficit) was proportional to the estimated annual N_2_ fixation rate in lakes. Mean differences in all variables were assessed for direct (treatment or year) and interactive (treatment and year) effects using linear mixed‐effects (LME) models in the R (Version 4.2.3, R Core Team, 2023). Further details can be found in Appendix S3.

## RESULTS

We explored the direct and interactive effects of year and treatment on water column TN:TP, chl *a*, *F*_fix_, and N_I_ which are shown for the three sampling years by N:P treatments (Table 1). There was a statistically significant interaction between year and treatment for TN:TP (LME ANOVA, χ^2^_8_ = 56.74, *p* < 0.001), chl *a* (LME ANOVA, χ^2^_8_ = 59.82, *p* < 0.001), N_I_ (LME ANOVA, χ^2^_8_ = 47.19, *p* < 0.001), and *F*_fix_ (LME ANOVA, χ^2^_6_ = 59.43, *p* < 0.001). However, these differences were not consistent among treatments and showed no monotonic trend. Additionally, the effect of year on these variables was minor compared with the relatively large effects observed among treatments.

**TABLE 1:** Geometric mean and SD of water column TN:TP and mean ± SD chlorophyll *a* (chl *a*), N imbalance relative to P (N_I_), and the fractional contribution of fixed N_2_ in seston (*F*_fix_), for each year sampled within each treatment.

Treatment/year	TN:TP (molar)	Chl *a* (μg L^−1^)	N_I_ (μg L^−1^)	*F*_fix_
Control (Pond)				
2019	48 [1.6]^A^	8.7 ± 5.9^A^	206 ± 2120^A^	N/A
2020	60 [1.6]^B^	6.4 ± 6.3^A^	498 ± 2310^A^	N/A
2021	47 [1.4]^A^	4.4 ± 1.9^B^	−4 ± 175^A^	N/A
Low N (2.2)				
2019	17 [2.4]^A^	18 ± 14^A^	−1590 ± 2240^A^	0.53 ± 0.24^A^
2020	29 [2.6]^B^	17 ± 18^A^	176 ± 3880^B^	0.74 ± 0.12^B^
2021	10 [2.1]^C^	16 ± 19^A^	−2510 ± 1890^C^	0.62 ± 0.09^A^
Med‐low N (16)				
2019	67 [1.9]^A^	21 ± 16^A^	361 ± 2480^A^	0.36 ± 0.21^A^
2020	63 [1.9]^AB^	21 ± 24^A^	367 ± 2290^A^	0.42 ± 0.18^B^
2021	48 [2.1]^B^	27 ± 28^A^	−380 ± 1130^B^	0.32 ± 0.16^A^
Med‐high N (55)				
2019	180 [2.1]^A^	37 ± 28^A^	3730 ± 3120^A^	0.17 ± 0.17^A^
2020	130 [2.2]^B^	65 ± 43^B^	2550 ± 2880^B^	0.09 ± 0.04^A^
2021	130 [1.6]^B^	52 ± 39^B^	3420 ± 2120^AB^	0.04 ± 0.05^B^
High N (110)				
2019	350 [2.2]^A^	39 ± 34^A^	8730 ± 5830^AB^	0.28 ± 0.21^A^
2020	360 [2.4]^A^	62 ± 51^B^	7660 ± 6430^A^	0.08 ± 0.06^B^
2021	290 [2.0]^A^	71 ± 41^B^	10,500 ± 5290^B^	0.02 ± 0.03^C^

*Note*: Letters indicate significant differences (*p* < 0.05) between years within each treatment based on linear mixed‐effects models and multiple comparison tests.

Abbreviations: N/A, not applicable; TN, total nitrogen; TP, total phosphorus.

**FIGURE 2:**
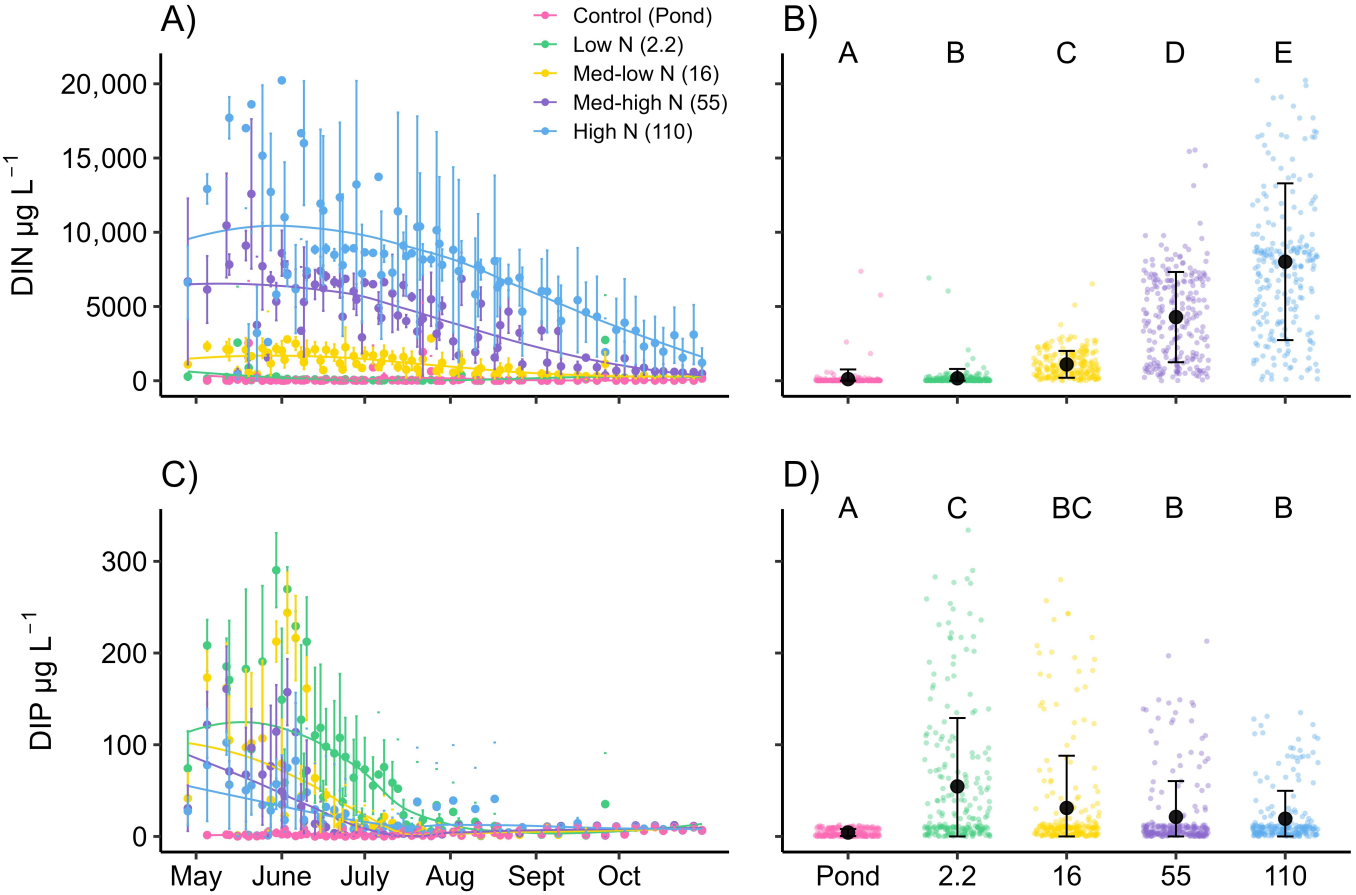
Treatment mean ± SD per sample event with corresponding LOESS lines for dissolved inorganic nitrogen (DIN, A) and dissolved inorganic phosphorus (DIP, C) demonstrate temporal changes throughout the sampling season. Data from all 3 years are plotted using Julian date. The mean ± SD for each treatment across all years for DIN (B) and DIP (D) are represented by the black dot and error bars and are plotted on top of raw data points.

Dissolved inorganic nutrients in the mesocosms exhibited an extremely consistent seasonal pattern among the 3 years of mesocosm fertilization resulting in clear treatment differences (Figure 2). Both DIN and DIP concentrations were greatest in spring when weekly fertilization rates were highest but decreased through the summer as fertilization rates slowed. DIN concentrations increased proportionally to N fertilization (LME ANOVA, *F*_4,10_ = 384.15, *p* < 0.001) with concentrations ranging from 111 ± 651 μg L^−1^ in the control ponds to 167 ± 626 μg L^−1^ in the low N treatment, 1103 ± 903 μg L^−1^ in the medium‐low N treatment, 4286 ± 3039 μg L^−1^ in the medium‐high N treatment, and 8016 ± 5279 μg L^−1^ in the high N treatment. DIP concentrations also varied among treatments (LME ANOVA, *F*_4,10_ = 60.21, *p* < 0.001), even though P fertilization was identical for all the treatments. DIP concentrations were lowest in the control ponds (4 ± 4 μg L^−1^) and greatest in the low N treatment (55 ± 75 μg L^−1^), while moderate mean DIP concentrations (19 ± 30 – 31 ± 57 μg L^−1^) were observed in the other treatments.

**FIGURE 3:**
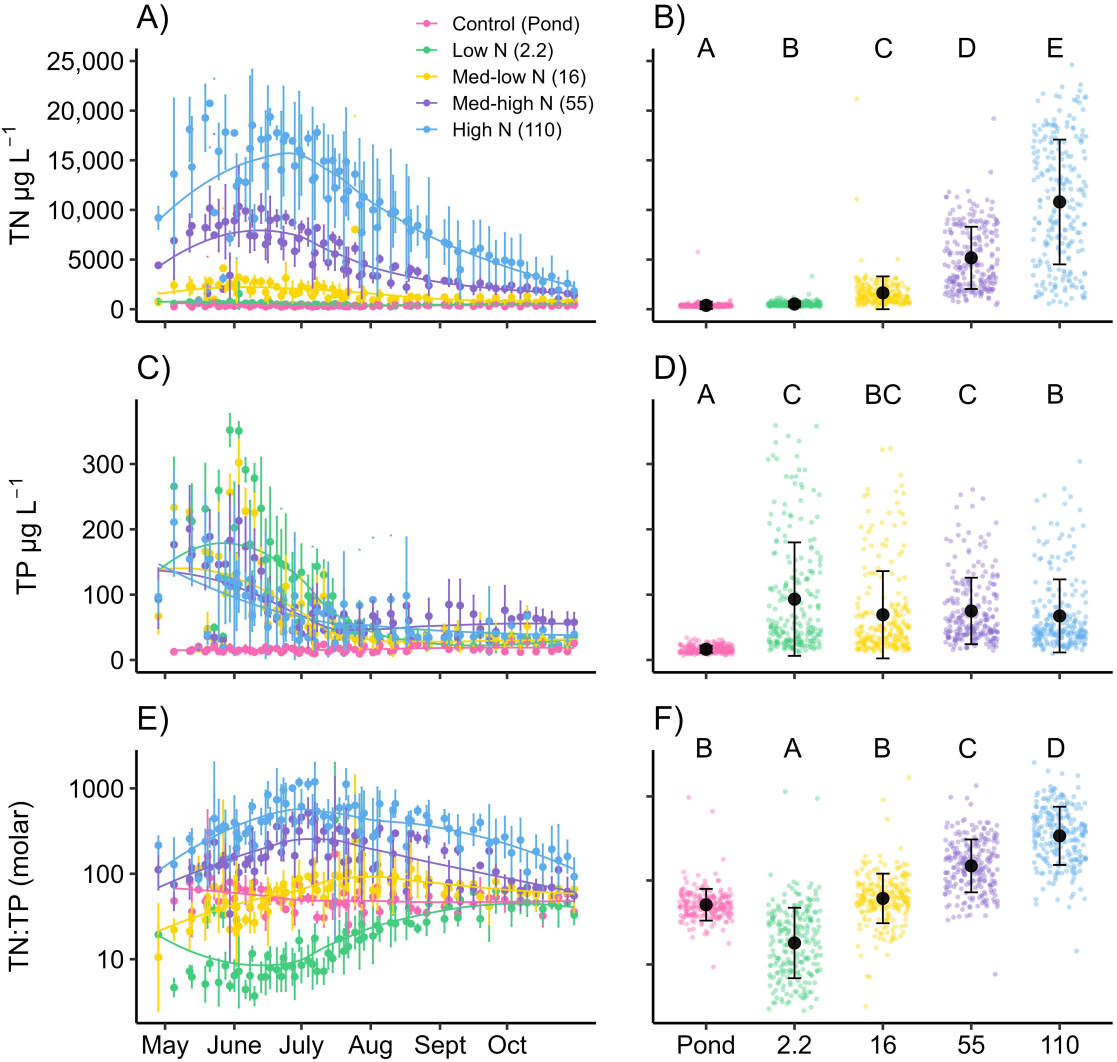
Treatment mean ± SD per sample event with corresponding LOESS lines for total nitrogen (TN, A) total phosphorus (TP, C) and geometric mean and SD of water column TN:TP water column TN:TP (E) demonstrate temporal changes throughout the sampling season. Data from all 3 years are plotted using Julian date. The mean ± SD for each treatment across all years for TN (B) TP (D) and geometric mean and SD of water column TN:TP water column TN:TP (F) are represented by the black dot and error bars and are plotted on top of raw data points. TN:TP plots (E, F) have a log scale y axis.

TN and TP concentrations were greater than DIN and DIP concentrations but exhibited a similar pattern seasonally, among years, and among treatments, which resulted in clear patterns for the TN:TP ratio (Figure 3). TN concentrations increased proportionally to N fertilization (LME ANOVA, *F*_4,10_ = 351.36, *p* < 0.001), with concentrations ranging from 388 ± 374 μg L^−1^ in the control ponds, 548 ± 420 μg L^−1^ in the low N treatment, 1649 ± 1657 μg L^−1^ in the medium‐low N treatment, 5171 ± 3135 μg L^−1^ in the medium‐high N treatment, and 10,799 ± 6284 μg L^−1^ in the high N treatment. TP concentrations also varied among treatments (LME ANOVA, *F*_4,10_ = 159.54, *p* < 0.001) even though P fertilization was identical across all treatments. TP concentrations were lowest in the control ponds (16 ± 5 μg L^−1^) and greatest in the low N treatment (93 ± 87 μg L^−1^), with minor differences observed among the other treatments. These patterns in TN and TP concentrations yielded a consistent seasonal pattern in TN:TP across all 3 years. Fertilization resulted in a clear seasonally divergent response in TN:TP among treatments in late spring and early summer, with TN:TP first increasing at higher N fertilization levels before decreasing later in summer. The opposite pattern occurred in the lowest N fertilization levels with TN:TP decreasing in late spring early summer before increasing later in the year. Despite the initial divergence and eventual near convergence on an annual basis, TN:TP was always greatest in the high N fertilization (Table 1, Figure 3, LME ANOVA, *F*_4,10_ = 94.16, *p* < 0.001). Measured TN:TP values were notably greater than target N:P fertilization ratios, likely due to a pronounced seasonal trend, where by late July, TP concentrations in all treatments had decreased to a range between the detection limit and 50 μg L^−1^ (Figure 3).

**FIGURE 4:**
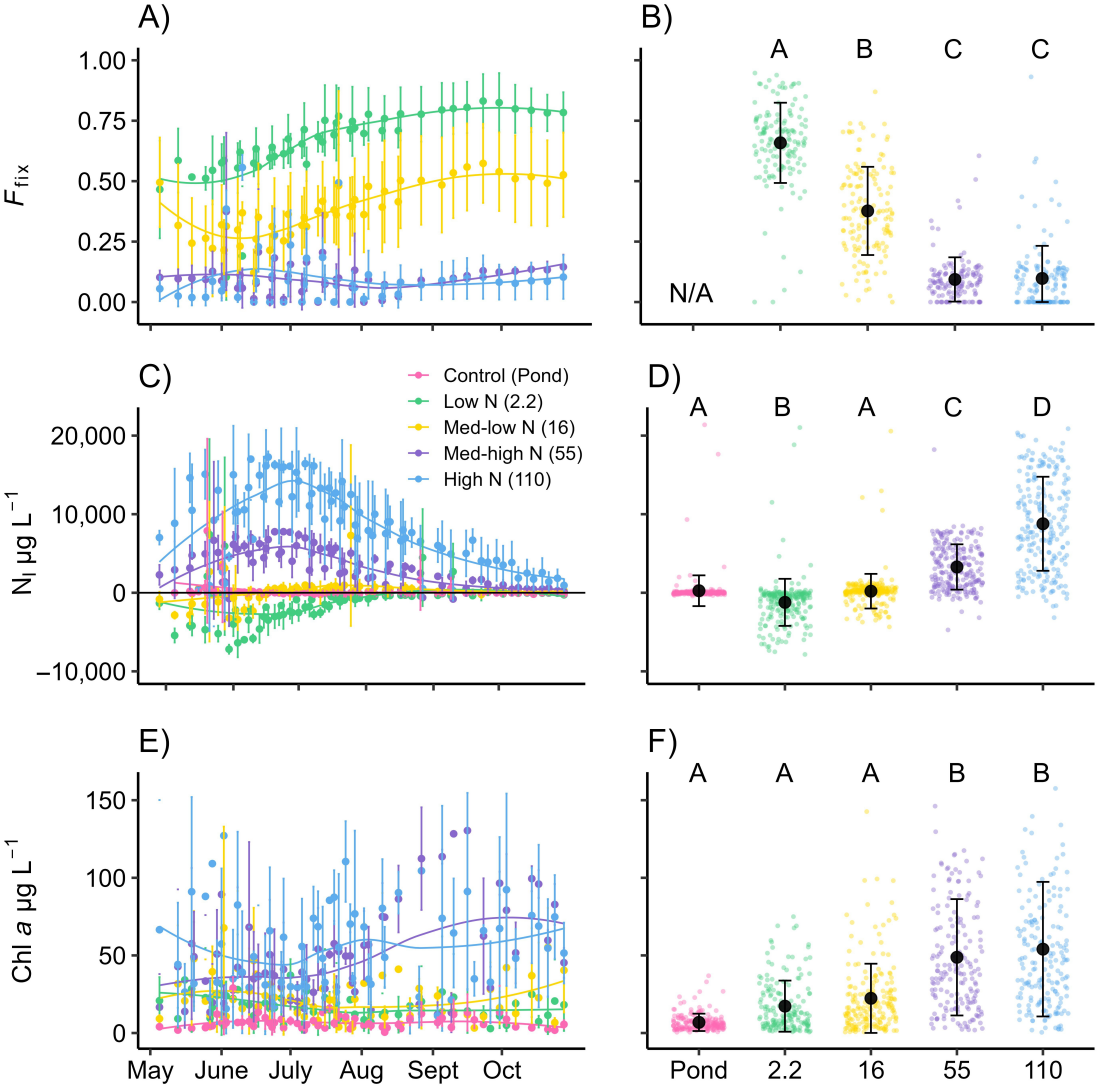
Treatment mean ± SD per sample event with corresponding LOESS lines for fractional contribution of fixed N_2_ in seston (*F*_fix_, A) N imbalance relative to P (N_I_, C) and chlorophyll *a* (chl *a*, E) demonstrate temporal changes throughout the sampling season. The black *y* = 0 line (C) indicates an N_I_ of 0. Values above the line indicate the N surplus relative to P and values below the line indicate the N deficit relative to P. Data from all 3 years are plotted using Julian date. The mean ± SD for each treatment across all years for *F*_fix_ (B), N_I_ (D), and chl *a* (F) are represented by the black dot and error bars and are plotted on top of raw data points. N/A, not applicable.

Similar to the nutrient concentrations, *F*_fix_ and N_I_ both exhibited consistent seasonal patterns that repeated among years (Figure 4). *F*_fix_ tended to increase throughout the summer period, and the pattern was most obvious in the low and medium‐low N treatments where *F*_fix_ was greatest (LME ANOVA, *F*_3,8_ = 45.81, *p* < 0.001). The seasonal pattern in N_I_ was also extremely strong but more complicated than other variables. The control ponds and the medium‐low N treatment (N:P = 16) exhibited similar N_I_ (258 ± 1958 vs. 201 ± 2204 μg L^−1^, respectively), even though TN (388 ± 374 vs. 1649 ± 1657 μg L^−1^, respectively) and TP (16 ± 5 vs. 69 ± 67 μg L^−1^, respectively) concentrations were significantly different. In the low N treatment, N_I_ decreased (i.e., the N deficit became stronger) from late spring through early summer before steadily increasing from mid to late summer, which resulted in a mean N_I_ that was the lowest among all treatments (LME ANOVA, *F*_4,10_ = 87.47, *p* < 0.001). Conversely, N_I_ in the medium‐high and high N treatments increased in late spring and early summer before steadily decreasing from mid to late summer, which resulted in mean N_I_ that was greater than the control ponds, low and medium‐low N treatments. Phytoplankton biomass measured as chl *a* showed no consistent seasonal pattern like the other measurements. However, there were clear treatment effects (LME ANOVA, *F*_4,10_ = 21.34, *p* < 0.001) with chl *a* being greatest in the medium‐high and high N treatments and lowest in the control ponds, low and medium‐low N treatments (Figure 4). Although chl *a* variability was greater in the low and medium‐low N treatments than the controls, there were no differences in chl *a* values among these treatments.

## DISCUSSION

The objective of this study was to test the effect of variable stoichiometry on the timescale required for eutrophic lakes to transition from proximate N limitation to ultimate P limitation. Our study was unique because we tested multi‐annual patterns in fully replicated mesocosms simulating ecosystem‐scale biogeochemical dynamics. Hundreds of previous studies have tested nutrient and primary production relationships with short‐term microcosm experiments (Elser et al., 2007) that do not capture ecosystem‐scale dynamics, or with whole‐lake experiments that have limited experimental range and lack replication (Wurtsbaugh et al., 2019). We tested three alternative hypotheses for shallow lakes experiencing high P loading with variable N stoichiometry: (1) The shift from N limitation to P limitation occurs quickly (weeks to months) due to seasonal accumulation of N relative to P; (2) the shift from N limitation to P limitation occurs at a moderate pace (in year[s]) due to interannual accumulation of N relative to P; or (3) the shift from N limitation to P limitation occurs slowly (in decades) or not at all due to no interannual accumulation of N relative to P. We observed no multi‐annual trends that would indicate fixed N accumulation across years but found highly consistent treatment effects on phytoplankton biomass, N_2_ fixation, and N imbalance relative to P. These results suggest that in the two lowest N treatments, N_2_ fixation was unable to fully alleviate N deficiency to allow for equal phytoplankton biomass with medium‐high and high N treatments, leading us to reject H1. While we observed seasonal trends in TN, TP, and TN:TP ratio suggesting seasonally converging TN:TP across treatments, there remained an order of magnitude difference in TN:TP among treatments at the end of each growing season, and there was no evidence of fixed N accumulation between years, leading us to reject H2. Our experimental evidence best supports H3, that shallow eutrophic lakes transition from proximate N limitation to ultimate P limitation extremely slowly (much greater than 3 years), if at all, because fixed N did not measurably accumulate relative to P, nor was there evidence that N_2_ fixation was slowing in response to accumulating N.

N_2_ fixation can compensate for the N loading deficit relative to P in some lake conditions (Scott et al., 2008, 2019), which presumably would make primary production proportional to P availability (Higgins et al., 2018). However, external P inputs predict phytoplankton biomass best when reactive N inputs are proportionally greater than P (Scott & McCarthy, 2011). Indeed, we observed significantly less phytoplankton biomass in the low and medium‐low N treatments compared with the medium‐high and high N treatments, which notably also corresponded with the pattern in measured primary production reported for 2019 (Kelly et al., 2021). Furthermore, we confirmed that the substantial fixed N contributing to the N supply in the two lowest N treatments could not compensate for observed N deficits on primary productivity. N_2_ fixation is an energetically expensive process in which 16 ATP are required to convert one N_2_ molecule into two ammonium molecules (Flores & Herrero, 2005). Many cyanobacteria common to shallow eutrophic lakes develop specialized anaerobic cells (heterocytes) that facilitate N_2_ fixation, but this creates a demographic cost of removing 10%–15% of cyanobacterial biovolume from cells that would otherwise be contributing chemical energy through photosynthesis (Grover et al., 2019). N_2_ fixation can also be constrained by numerous abiotic factors such as light availability (Scott & Grantz, 2013), water column mixing (Moisander et al., 2002), oxygen supersaturation (Paerl, 2018), warm water temperatures (Kramer & Gobler, 2023), and micronutrient availability (Glass et al., 2010). Cyanobacterial N_2_‐fixers are also susceptible to zooplankton grazing (Hambright et al., 2007), and their vulnerability may be magnified by the growth rate cost in forming heterocytes for N_2_ fixation (Grover et al., 2022). These known constraints manifest in a growth trade‐off with N_2_ fixation in cyanobacteria (Osburn et al., 2021), which may explain constraints on primary production even if the N pool comes into balance with P stoichiometrically (Baker et al., 2018; Boatman et al., 2018; Thiel & Pratte, 2014).

While we did not specifically measure N and P loss estimates, we found no measurable N accumulation over the 3‐year study period. Thus, our findings support the idea that annual reactive N gains from N_2_ fixation are less than annual N removal via burial or denitrification in most eutrophic lakes (Grantz et al., 2014; Scott et al., 2019). An N removal rate exceeding N gain would prevent ecosystem‐scale N accumulation. Oligotrophication of the Laurentian Great Lakes due to P declines has resulted in reactive N accumulation due to organic matter and redox constraints on denitrification (Finlay et al., 2013). In contrast, the N loss due to increased organic matter and favorable redox conditions must also occur in lakes experiencing eutrophication (Scott et al., 2019). Prior results from our mesocosms support this hypothesis. N_2_ gas saturation was monitored in the mesocosms and demonstrated that net N gain (i.e., N_2_ fixation rates > denitrification rates) only occurred in the lowest N treatment while balanced gains and losses were observed at N:P supply ratio of 16, and net N loss (i.e., denitrification rates > N_2_ fixation rates) occurred in the high N treatments (Taylor et al., 2023). The lack of net N gain at 16 N:P supply suggests that eutrophic lakes with N:P inputs that induce strict N limitation (N:P < 20; Graeber et al., 2024; Guildford & Hecky, 2000) do not import more N into the lake via N_2_ fixation than what is exported to the atmosphere via denitrification. Collectively, this evidence points toward a fundamental biogeochemical mechanism that constrains the potential for a transition from proximate N limitation to ultimate P limitation in eutrophic lakes.

Watersheds with variable land use can cause highly variable stoichiometric inputs to lakes (Hayes et al., 2015; Vanni et al., 2011), and our experimental treatments account for this range. The frequency and magnitude of disturbance regimes (most frequently storm events) control nutrient loading from watersheds to lakes (Carpenter et al., 2022) and therefore control the nutrient stoichiometry fueling primary productivity (Kelly et al., 2019). Internal nutrient loading of stored P (and N, if not denitrified) from the sediments can also influence water column stoichiometry providing a positive feedback loop in eutrophic lakes (Burger et al., 2008). For example, in shallow eutrophic lakes that have a history of agricultural nutrient loading, sediments may generate internal nutrient loads proportional to external watershed loads on an annual basis (Nifong et al., 2022). As eutrophic semi‐closed systems, our mesocosms stored P highly efficiently. Internal N and P loading was not measured directly in our experiment, but N_I_ tracked our seasonal fertilization, which indicated the strong importance of external inputs in controlling water column stoichiometry.

Our experimental approach was designed to strike a balance between replication and realism (Schindler, 1998). The open top and bottom mesocosms allowed a complete air‐water‐sediment connection necessary to test the effects of N transformations on ecosystem stoichiometry and productivity (Scott & McCarthy, 2010). Additionally, our seasonal fertilization regime was designed to reflect natural spring and summer precipitation patterns common to North America (Royer et al., 2006), which serve as the major source of nutrient supply to lakes. Nevertheless, some realism was sacrificed that may have influenced our results. We assumed that microbial growth on the mesocosm walls minimally impacted nutrient concentrations and primary productivity due to the large volume to surface area ratio in our 3‐m diameter mesocosms. Our mesocosms likely also maximized export of material from the water column to sediments by creating an extremely stable water column with little or no advection, particularly since our mesocosms were in small shallow lakes that develop little or no wave action in response to high wind. Importantly, the geographic setting of our experiment in northern Mississippi make our results more applicable to shallow lakes in plains and lowland regions rather than deep lakes in highland and mountainous regions (Zhou et al., 2022). These experimental limitations represented trade‐offs between observing multi‐annual patterns in a truly replicated design versus a realistic scale for whole‐ecosystem experimentation.

Our results show that the known ecological constraints on N_2_ fixation (Grover et al., 2019, 2022) and the potential energetically favorable losses of available N by denitrification (Grantz et al., 2014) create a mechanism for extending the duration of N limitation to multiple years or longer in shallow eutrophic lakes where nutrient inputs favor N limitation. Thus, N gains that shift ecosystem stoichiometry in response to P fertilization occur extremely slowly, if at all, in shallow eutrophic lakes. We found no evidence to support N accumulation relative to P between years, even under extremely low N fertilization regimes. Although fixed N may accumulate in less productive oligotrophic lakes, eutrophic lakes experience the opposite because fixed N inputs do not exceed losses due to N burial or denitrification. Our results not only support eutrophication management strategies that focus on reduction of both P and N (Andersen et al., 2020; Paerl et al., 2016) to mitigate the negative effects of nutrient enrichment, but also have implications for theoretical development in ecosystem stoichiometry for freshwater, marine, and terrestrial environments.

## AUTHOR CONTRIBUTIONS

J. Thad Scott, Jason M. Taylor, Patrick T. Kelly, and Isabelle M. Andersen contributed to the study design. Isabelle M. Andersen, Alexa K. Hoke, Patrick T. Kelly, and Jason M. Taylor collected data and performed laboratory analyses. Isabelle M. Andersen, Jason M. Taylor, and Caleb J. Robbins performed statistical analyses. Isabelle M. Andersen and J. Thad Scott wrote the first draft of the manuscript, and all authors contributed to revisions. All authors read and approved the final manuscript.

## CONFLICT OF INTEREST STATEMENT

The authors declare no conflicts of interest.

## Supporting information


Appendix S1.

Appendix S2.

Appendix S3.

## Data Availability

Data and code (Andersen et al., 2024) are available in Figshare at https://doi.org/10.6084/m9.figshare.25739277.
