# Supplementary material for: Nitrogen fixation may not alleviate stoichiometric imbalances that limit primary production in eutrophic lake ecosystems
Source: Ecology. 2025 Jan 24;106(1):e4516. doi: 10.1002/ecy.4516 (PMC11758711; doi:10.1002/ecy.4516)
Supplement: Supplementary file 1 — Appendix S1. [file ECY-106-e4516-s001.pdf]

**Appendix S1**

Journal: Ecology

Nitrogen fixation may not alleviate stoichiometric imbalances that limit primary production in eutrophic lake ecosystems

Isabelle M. Andersen, Jason M. Taylor, Patrick T. Kelly, Alexa K. Hoke, Caleb J. Robbins, J. Thad Scott

*Study Site and Experimental Design*

Experiments were conducted in ponds at the University of Mississippi Biological Station. Surface water runoff inputs into experimental ponds are excluded by ditches and berms and the primary water source is precipitation with excess water draining through PVC standpipes that are set to prevent flooding. The ponds are mesotrophic with average total N (TN), total P (TP), and chlorophyll-a (chl-a) concentrations of 388±374 μg $L^{-1}$, 16±5 μg $L^{-1}$, and 7±5 μg $L^{-1}$ (mean±SD), respectively. Ponds were drained before mesocosm installation to ensure that mesocosm bases were securely anchored into pond sediments then refilled with water from surrounding ponds.

Mesocosms were accessed via a floating walkway connected to the shore. A mesocosm with each experimental N level was positioned randomly in each of three ponds in a randomized block design. Mesocosms were dosed with 1.3 g P $m^{-2}$ $y^{-1}$ which is an approximate median for a range of oligotrophic to hypereutrophic lakes globally (Table S1). Nitrogen fertilizer was added proportionally to P at the following molar ratios: 2.2, 16, 55, 110.

**Table S1**. Measured annual external loading rates of P (g $m^{-2}$ $y^{-1}$) of lakes compared to P loading rates for our experiment. Most loading rates are from Nürnberg 1984 unless otherwise cited.

| Lake (Country) | Annual external loading of P (g $m^{-2}$ $y^{-1}$) |
|---|---|
| Superior (CA, US) | 0.03 |

| | |
|---|---|
| Clear (CA) | 0.04 |
| Tahoe (US) | 0.042 |
| Huron (CA, US) | 0.07 |
| Michigan (US) | 0.1 |
| Aegeri (CH) | 0.16 |
| Chemung (US) | 0.22 |
| Turler (CH) | 0.3 |
| Chautauqua (US) | 0.34 |
| Washington (US) | 0.46 |
| Lake 227 (CA) (Schindler 2008) | 0.48 |
| Cayuga (US) | 0.55 |
| Hallwiler (CH) | 0.55 |
| Esrom (DK) | 0.6 |
| Shawaga (US) | 0.65 |
| Wedington (US) (Grantz 2014) | 0.65 |
| Ontario (CA, US) | 0.68 |
| Harriet (US) | 0.71 |
| Burr Oak (US) (Vanni et al. 2011) | 0.79 |
| Mesocosm Experiment | 1.3 |
| Zurich (CH) | 1.32 |
| Saratoga (US) | 1.6 |
| Baldegger (US) | 1.75 |
| Elmdale (US) (Grantz et al. 2014) | 3.2 |

| | |
|---|---|
| Maggiore (CH, IT) | 3.39 |
| Waco (US) (Scott et al. 2008) | 3.49 |
| Pleasant Hills (US) (Vanni et al. 2011) | 4.25 |
| Acton (US) (Vanni et al. 2011) | 4.74 |
| Fayetteville (US) (Grantz et al. 2014) | 7.1 |
| Bergundasjöen (SE) | 8.8 |

In week one each year, we dosed with 2.5 g P and 2.5 g N, 18.1 g N, 62.6 g N, or 125.2 g N, respectively, and then decreased these dosing amounts each week by approximately 33%. This pattern was repeated in each year to simulate the spring/early summer high rates of external nutrient inputs and the cessation of inputs through summer as precipitation declines seasonally.

**References**

Grantz, E.M., B.E. Haggard, and J.T. Scott. 2014. "Stoichiometric imbalance in rates of nitrogen and phosphorus retention, storage, and recycling can perpetuate nitrogen deficiency in highly-productive reservoirs." *Limnology and Oceanography* 59: 2203–2216.

Nürnberg, G. K. 1984. "The prediction of internal phosphorus load in lakes with anoxic hypolimnia" *Limnology and Oceanography 29*: 111-124.

Schindler, D.W., R.E. Hecky, D.L. Findlay, M.P. Stainton, B.R. Parker, M.J. Paterson, K.G. Beaty, M. Lyng and S. Kasian. 2008. "Eutrophication of lakes cannot be controlled by reducing nitrogen input: results of a 37-year whole-ecosystem experiment." *Proceedings of the National Academy of Sciences* 105: 11254-11258.

Scott, J.T., M.J. McCarthy, W.S. Gardner, and R.D. Doyle. 2008. "Denitrification, dissimilatory nitrate reduction to ammonium, and nitrogen fixation along a nitrate concentration gradient in a created freshwater wetland." *Biogeochemistry* 87: 99–111.

Vanni, M.J., W.H. Renwick, A.M. Bowling, M.J. Horgan, and A.D. Christian. 2011. "Nutrient stoichiometry of linked catchment-lake systems along a gradient of land use." *Freshwater Biology* 56: 791–811.
