# Supplementary material for: Nitrogen fixation may not alleviate stoichiometric imbalances that limit primary production in eutrophic lake ecosystems
Source: Ecology. 2025 Jan 24;106(1):e4516. doi: 10.1002/ecy.4516 (PMC11758711; doi:10.1002/ecy.4516)
Supplement: Supplementary file 2 — Appendix S2. [file ECY-106-e4516-s002.pdf]

Appendix S2

Journal: Ecology

Nitrogen fixation may not alleviate stoichiometric imbalances that limit primary production in eutrophic lake ecosystems

Isabelle M. Andersen, Jason M. Taylor, Patrick T. Kelly, Alexa K. Hoke, Caleb J. Robbins, J. Thad Scott

N Isotopic Labeling

Mesocosms were isotopically labeled with ^{15}N (as $Na^{15}NO_3$) to estimate the relative inputs of fertilizer and atmospheric N. Isotopic labeling was proportional to the fertilization level so that the target value of isotopic labeling across all experimental units was approximately 100‰ $\delta^{15}N$.

Isotopic Mixing Model

The fractional contribution of fixed N_2 in seston (F_{fix}) was estimated using a two-source mixing model:

$$\delta^{15}N_{seston} = (F_{fix})(\delta^{15}N_{fix}) + (F_{added})(\delta^{15}N_{added}) \qquad \text{(Equation S1)}$$

where $\delta^{15}N_{seston}$ is the measured isotopic composition of seston, F_{fix} and F_{added} are the unknown fractional contributions of N_2 fixation and N fertilizer to the seston, and $\delta^{15}N_{fix}$ and $\delta^{15}N_{added}$ are the isotopic signatures of fixed N_2 and fertilizer N, respectively. $\delta^{15}N_{fix}$ was assumed to be 0‰ because N_2 gas was used as the reference standard in isotopic analysis. The $\delta^{15}N_{added}$ was targeted at 100‰, but we accounted for a maximum uptake fractionation of ~15‰ (Bauersachs et al. 2009) and assumed a value of 85‰ for $\delta^{15}N_{added}$. Assuming that N_2 fixation and fertilizer N were the two major sources of N to seston, the sum of F_{fix} and F_{added} equals one. Substituting 1-

F_{fix} for F_{added} and including the zero assumption for $\delta^{15}N_{fix}$, the two-source mixing model can be solved for F_{fix} as:

$$F_{fix} = 1 - \frac{\delta^{15}N_{seston}}{\delta^{15}N_{added}} \qquad \text{(Equation S2)}$$

Thus, F_{fix} is a variable that ranges from 0-1 with values representing the proportion of N in seston that was derived from N_2 fixation.

References

Bauersachs, T., S. Schouten, J. Compaoré, U. Wollenzien, L.J. Stal, and J.S. Sinninghe Damsteé. 2009. "Nitrogen isotopic fractionation associated with growth on dinitrogen gas and nitrate by cyanobacteria." *Limnology and Oceanography* 54: 1403–1411.
