# Supplementary material for: Nitrogen fixation may not alleviate stoichiometric imbalances that limit primary production in eutrophic lake ecosystems
Source: Ecology. 2025 Jan 24;106(1):e4516. doi: 10.1002/ecy.4516 (PMC11758711; doi:10.1002/ecy.4516)
Supplement: Supplementary file 3 — Appendix S3. [file ECY-106-e4516-s003.pdf]

Appendix S3

Journal: Ecology

Nitrogen fixation may not alleviate stoichiometric imbalances that limit primary production in eutrophic lake ecosystems

Isabelle M. Andersen, Jason M. Taylor, Patrick T. Kelly, Alexa K. Hoke, Caleb J. Robbins, J. Thad Scott

Statistical Analyses

For each response variable, we selected from a suite of models including combinations of random intercepts, variance covariate weighting, and autocorrelation structures following Zuur et al. (2009). Visualizations of residuals from a base linear model without any modeled residual structure showed strong temporal autocorrelation and heteroskedasticity. We therefore clustered observations within mesocosm with a random intercept. We also modeled temporal autocorrelation in the residuals for each mesocosm as a first-order autoregressive function using corAR1() in package *nlme* (Pinheiro et al. 2020). Finally, we accounted for covariate heteroskedasticity as a function of treatment and year with the function varIdent() in package *nlme* (Pinheiro et al. 2020). We selected the final model for inference based on AIC comparisons, and confirmed assumptions of normality, homoskedasticity, and independence by visualizing the standardized residuals. In some cases, temporal autocorrelation remained in the residuals, but was substantially reduced from the base model without correlation structures. We conducted omnibus tests on each final model using F tests based on Type I sums of squares (for additive models) and in Wald chi-square tests based on Type II sums of squares (for interactive models) using the function Anova() in the package *car*. We used the *emmeans* package to estimate and test Tukey post-hoc contrasts between treatments and years, or overall treatment effect (i.e.,

treatment effect averaged across years) (Lenth et al. 2022). TN:TP values were logged for calculation of geometric mean, standard deviation, and statistical analyses (Isles 2020). Differences were significant at a level of $p < 0.05$. We visualized seasonal trends of response variables across all sampling dates for each treatment using locally estimated scatterplot smoothing (LOESS) lines. All analyses were done using R (Version 4.2.3, R Core Team 2023).

References

Isles, P.D. 2020. “The misuse of ratios in ecological stoichiometry.” *Ecology* 101: 03153.

Lenth, R.V., P. Buerkner, I. Giné-Vázquez, M. Herve, M. Jung, J. Love, F. Miquez, H. Riebl, H. Singmann, @ R Core Team. 2022. “emmeans.” R package version 1.8.3. https://cran.rproject.org web/packages/emmeans/ index.html.

Pinheiro, J., D. Bates, S. DebRoy, D. Sarkar, @ R Core Team. 2020. “nlme: linear and nonlinear mixed effects models.” R package version 3.1-150. https:// CRAN.r- project. org/ package=nlme.

R Core Team. 2023. *R: a language and environment for statistical computing*. Vienna, Austria: R Foundation for Statistical Computing.

Zuur, A.F., E.N. Ieno, N. Walker, A.A. Saveliev, and G.M. Smith. 2009. “Mixed effects models and extensions in ecology with R.” Statistics for Biology and Health. Springer, New York, NY.
